# Epithelial Na^+^ Channel (ENaC) Formed by One or Two Subunits Forms Functional Channels That Respond to Shear Force

**DOI:** 10.3389/fphys.2020.00141

**Published:** 2020-03-17

**Authors:** Jan-Peter Baldin, Daniel Barth, Martin Fronius

**Affiliations:** ^1^Department of Physiology, School of Biomedical Sciences, University of Otago, Dunedin, New Zealand; ^2^Institute of Physiology, Rheinisch-Westfälische Technische Hochschule Aachen University, Aachen, Germany; ^3^HeartOtago, University of Otago, Dunedin, New Zealand

**Keywords:** epithelial sodium channel, ENaC, homotrimer, mechanosensitive, shear force, flow, amiloride-sensitive current

## Abstract

Canonical epithelial sodium channels (ENaCs) are heterotrimers formed by α, β, and γ ENaC subunits in vertebrates and belong to the Degenerin/ENaC family of proteins. Proteins from this family form mechanosensitive channels throughout the animal kingdom. Activity of canonical ENaC is regulated by shear force (SF) mediating Na^+^ absorption in the kidney and vascular tone of arteries. Expression analysis suggests that non-canonical ENaC, formed by single or only two subunits, exist in certain tissues, but it is unknown if these channels respond to SF. α, β, γ, and δ ENaC subunits were expressed either alone or in combinations of two subunits in *Xenopus* oocytes. Amiloride-sensitive currents and the responses to SF were assessed using two-electrode voltage clamp recordings. With the exception of γ ENaC, all homomeric channels provided amiloride-sensitive currents and responded to SF applied via a fluid stream directed onto the oocytes. Channels containing two subunits were also activated by SF. Here, the presence of the γ ENaC subunit when co-expressed with α or δ augmented the SF response in comparison to the αβγ/δβγ ENaC. Overall, we provide evidence that non-canonical ENaC can form channels that respond to SF. This supports a potential function of non-canonical ENaC as mechanosensors in epithelial, vascular, and sensory cells.

## Introduction

The epithelial sodium channel (ENaC) proteins belong to the degenerin/ENaC (DEG/ENaC) superfamily of proteins (Mano and Driscoll, 1999). Channels formed by members of this family form mechanosensitive ion channels in various species across the animal kingdom (Mano and Driscoll, 1999). ENaC in vertebrates is expressed in epithelial tissues such as kidney, lung, and colon and plays a key role in maintaining salt and water homeostasis (Garty and Palmer, 1997; Rossier et al., 2015). Research in the last 20 years has accumulated evidence that ENaC is also expressed in non-epithelial cells, including vascular cells such as endothelial cells (Golestaneh et al., 2001; Kusche-Vihrog et al., 2008) and vascular smooth muscle cells (Drummond et al., 2008). There is also growing evidence that ENaC proteins in vascular cells may function as mechanosensors in both endothelial cells (Ashley et al., 2018), as well as smooth muscle cells (Drummond et al., 2004, 2008).

Canonical ENaC consists of three homologous subunits, known as α, β, and γ, that form a functional heteromultimeric channel (Canessa et al., 1994a). ENaC assembles with a 1:1:1 stoichiometry of α:β:γ subunits arranged in a counter-clockwise manner as revealed by cryo-electron microscopy (Noreng et al., 2018). Another subunit known as δ has been identified in humans (Waldmann et al., 1995), primates (Giraldez et al., 2007), and *Xenopus* (Wichmann et al., 2018) but is not expressed in mice or rats (Giraldez et al., 2012). Each individual ENaC subunit is made of 650–700 amino acids with a molecular weight of around 70–100 kDa, two transmembrane domains, short intracellular N- and C-termini, and a large extracellular-domain that consists of ~70% of the protein (Canessa et al., 1994a; Snyder et al., 1994; Noreng et al., 2018).

ENaC activity can be regulated by various factors, such as proteases (Vallet et al., 1997), pH (Ji and Benos, 2004), and mechanical force, such as shear force (SF) and pressure (Fronius and Clauss, 2008; Guo et al., 2016). SF is a frictional force primarily caused by the movement of particles (such as fluids) parallel to surfaces (e.g., luminal cell surfaces). Physiological SF caused by urine in kidney tubules was shown to activate ENaC (Satlin et al., 2001). Also, heterologous expressed αβγ ENaC was shown to be activated by SF (Carattino et al., 2004). Activation of ENaC by SF is due to an increased open probability (Althaus et al., 2007; Fronius et al., 2010; Wang et al., 2009). The mechanisms of how ENaC senses SF to induce opening of the pore are incomplete. Particularly, the role of certain subunits for the ability of ENaC to sense SF is unknown. This is of particular interest since the identification of individual ENaC subunits in certain tissues implies the existence of non-canonical ENaC that could be formed by different combinations of subunits, other than αβγ. This potential role of non-canonical ENaC as mechanosensors derives from a number of studies. For example, only expression of the γ ENaC subunit was identified in rat baroreceptors, indicating an important role for this subunit for mechanosensation and central blood pressure regulation (Drummond et al., 1998). In addition, β and γ ENaC, but not α ENaC, were identified in vascular smooth muscle cells of renal arteries, where they contribute to pressure-induced vasoconstriction (Drummond, 2007, 2012). Other studies provided evidence that β ENaC is essential for regulating renal arterial myogenic tone *in vitro* and *in vivo* (Chung et al., 2013). The findings in renal arteries highlight the importance of the β ENaC subunit for mechanosensation. Overall, there is evidence to support the hypothesis that non-canonical ENaC consisting of either one or combinations of two subunits could form functional channels and that these channels mediate mechanosensitive responses.

Therefore, the aim of this study was to determine whether individual ENaC subunits can form functional channels that respond to SF. To address this question, combinations of ENaC subunits were expressed as homotrimers or heterotrimers in *Xenopus* oocytes. Amiloride-sensitive currents were determined, and the response to SF was analyzed based on previously established methods (Althaus et al., 2007). Here, we show that the α, β, and δ ENaC subunits can form homomeric functional channels that show amiloride-sensitive currents and increased activation when exposed to SF, whereas γ ENaC cannot. Furthermore, we have evidence that β and γ ENaC have different modulatory roles for the response of SF when co-expressed with either α or δ.

## Materials and Methods

### Harvesting of *Xenopus laevis* Oocytes

Adult females of the South African clawed frog *Xenopus laevis* were purchased from eNASCO (Fort Atkinson, United States) and kept in an aquatic housing system (XenoPlus, Tecniplast). All procedures were conducted in accordance with the New Zealand Animal Welfare Act and were approved by the Animal Ethics Committee of the University of Otago (approval numbers: 114/13 and 83/16).

Oocytes were extracted by partial ovariectomy under anesthesia via MS-222 solution (tricaine methane sulfonate, Sigma-Aldrich) with a concentration of 1.3 g/L (buffered to a neutral pH). Extracted ovary lobes were immediately placed in culture oocyte ringers solution (CulORi) containing in mM: 90 NaCl, 1 KCl, 2 CaCl_2_, 5 HEPES, 2.5 Na^+^ pyruvate, 0.06 penicillin, 0.02 streptomycin, and in addition 50 μg/mL tetracycline, 100 μg/mL amikacin, 100 μg/mL ciprofloxacin, pH 7.4). For oocyte separation and removal of the follicle layer, the oocytes were incubated at room temperature for 90 min in collagenase (1.5 mg/mL dissolved in CulORi; Serva). Then, the oocytes were washed in Ca^2+^-free oocyte Ringer’s solution (containing in mM: 90 NaCl, 5 HEPES, 1 KCl, 1 EGTA, pH 7.4). Fully developed and healthy-looking stage V–VI oocytes (Dumont, 1972) were collected and stored individually in 96 well plates containing CulORi for further processing in an incubator at 17°C (Lab Companion).

### Heterologous Expression of ENaC in Oocytes of *Xenopus laevis*

For ENaC expression, the oocytes were microinjected with combinations of cRNA encoding human α (NCBI#: NM_001038.5), β (NCBI#: NM_000336.2), γ (NCBI#: NM_001039.3), and δ ENaC (NCBI#: NM_001130413.3). For corresponding control experiments, oocytes were injected with identical volumes of nuclease-free water. Microinjection was performed either by using a nanoject II Auto-Nanoliter Injector (Drummond Scientific) or a Roboinject (Multichannel Systems). Each oocyte was injected with 15 nl of cRNA that corresponded to a total cRNA amount of 0.24 ng (Table 1).

**TABLE 1:** Amount of cRNA injected per oocyte for the expression of ENaCs formed by one, two, or three subunit(s).

Channel	cRNA (ng/oocyte)
[mymaths]αβ[mymathe]γ ENaC	0.08 per subunit
[mymaths]αβ[mymathe]γ ENaC	0.08 per subunit
α ENaC	0.24
δ ENaC	0.24
β ENaC	0.24
γ ENaC	0.24
αβ ENaC	0.12 per subunit
αγ ENaC	0.12 per subunit
βγ ENaC	0.12 per subunit
δβ ENaC	0.12 per subunit
δγ ENaC	0.12 per subunit

Following injection, the oocytes were stored in 96 well plates filled with low Na^+^ solution (containing in mM: 10 NaCl, 80 NMDG (N-methyl-D-glucamine), 1 KCl, 2 CaCl_2_, 5 HEPES, 2.5 Na^+^ pyruvate, 0.06 penicillin, 0.02 streptomycin, and in addition 50 μg/mL tetracycline, 100 μg/mL amikacin, 100 μg/mL ciprofloxacin, pH 7.4) at 17°C in an incubator.

### Two-Electrode Voltage Clamp (TEVC)

The oocytes were placed in a custom-made flow chamber and perfused with oocyte Ringer’s solution (ORi, containing in mM: 90 NaCl, 1 KCl, 2 CaCl_2_, 5 HEPES, pH 7.4) driven by a pressurized perfusion system (ALA Scientific Instruments). The flow-chamber had a channel for perfusion, designed to allow a consistent application of SF to the oocyte’s surface and a rapid solution-exchange. The flow rate was adjusted to 2.4 mL/min and corresponded to a SF rate of about 0.2 dyn/cm^2^ on the oocytes surface. SF rates were calculated as described previously (Althaus et al., 2007). Transmembrane currents of oocytes voltage-clamped at a membrane potential of –60 mV (TURBO TEC-05 amplifier, NPI, Tamm, Germany) were digitized (PowerLab 4/35, ADInstruments) and recorded through LabChart (ADInstruments). Amiloride dose response curves were recorded at −60 mV via an automated TEVC system (Roboocyte2, Multichannel Systems) utilizing a 96 well plate design. Values from the dose-response traces were fitted with the Hill equation (variable Hill coefficient), and the determined IC_50_ concentrations for amiloride from individual recordings were used to calculate means for comparison between different channel compositions.

### Biotinylation Assay

Biotinylation assays (Biotin Z-link^®^ Sulfo-NHS-LC biotin, Thermo Fisher Scientific) were used to determine membrane expression of β ENaC in *Xenopus* oocytes. Therefore, 2 × 50 oocytes were injected with β ENaC and incubated for either 24 h or 48 h at 17*°*C. All biotinylation steps were performed at 4°C. Firstly, 2 × 50 oocytes were incubated in MBS buffer (containing in mM: 88 NaCl, 1 KCl, 2.4 NaHCO_3_, 0.8 MgSO_4_, 0.4 CaCl_2_, 10 HEPES, pH 7.4) for 30 min and subsequently washed three times in MBS. Fifty Oocytes were placed in biotinylation buffer (containing in mM: 10 mM triethanolamine, 150 mM NaCl, 2 mM CaCl2, pH 9.5) and incubated for 15 min on a platform shaker in presence and absence (control) of biotin (1 mg/mL). Biotinylation reaction was stopped via two washing steps in quench buffer (containing in mM: 192 mM glycine, 25 mM Tris-Cl, pH 7.5). Afterward, oocytes were incubated in quench buffer for 5 min, washed twice with MBS buffer and homogenized in lysis buffer (containing in mM: 83 NaCl, MgCl_2_, 10 HEPES, 1% Triton X-100, 1 tablet/10 mL protease inhibitor tablets, pH 7.9). The homogenate was then vortexed for 30 s and centrifuged by 5000 × *g* at 4°C for 10 min. Supernatants were transferred into 1.5 mL Eppendorf tubes and the first sample (whole cell) taken. The remaining whole cell sample of biotin (+) and control (−) was treated with the PNGaseF (New England Biolabs) for 1 h at 37°C. Fifty microliter Neutravadin^®^ Ultra Link^®^ beads (Thermo Fisher Scientific) were aliquoted into a 1.5 mL Eppendorf tube and washed with lysis buffer three times. PNGaseF-treated samples were transferred to the beads and shaken overnight at 4*°*C. To denaturate the samples, 5x Laemmli sample buffer (containing in%: 10 Tris, 5 SDS, 25 Glycerol, 0.8 bromphenol blue, 5 B-mercaptoethanol) was added, incubated for 10 min at 100°C before loaded on an 8% SDS-polyacrylamide gel. Protein samples were transferred electrophoretically to PVDF membranes. These membranes were then blocked for 1 h in TBS-T with 5% milk (containing in mM: 50 Tris, 150 NaCl with 0.1% Tween). Subsequently, the membranes were incubated overnight at 4°C within Anti-Flag HRP antibody (Sigma) diluted 1:2500 in TBS-T. The proteins were visualized with an enhanced chemiluminescent (ECL) (Amersham ECL Prime) and exposed to X-ray film (Radiographic Supplies).

### Statistics

Data are expressed as mean ± standard error of the mean (SEM). Numbers of experiments are presented as n. Oocytes from at least two animals were harvested and used for each experiment. Values from the electrophysiological recordings were collected from current recordings as indicated by arrows in figures and further analyzed with GraphPad Prism 6.07. Statistical comparisons were made using multiple comparisons one-way ANOVA, unpaired *t*-test, or paired Student’s *t*-test (indicated within corresponding figure legends). Statistical differences were indicated as following – ns *p ≥* 0.05; ^∗^*p <* 0.05; ^∗∗^*p <* 0.01; ^∗∗∗^*p <* 0.001; ^****^p < 0.0001.

## Results

### Homomeric α, β, and γ ENaCs Form Functional Amiloride-Sensitive Channels

**FIGURE 1:**
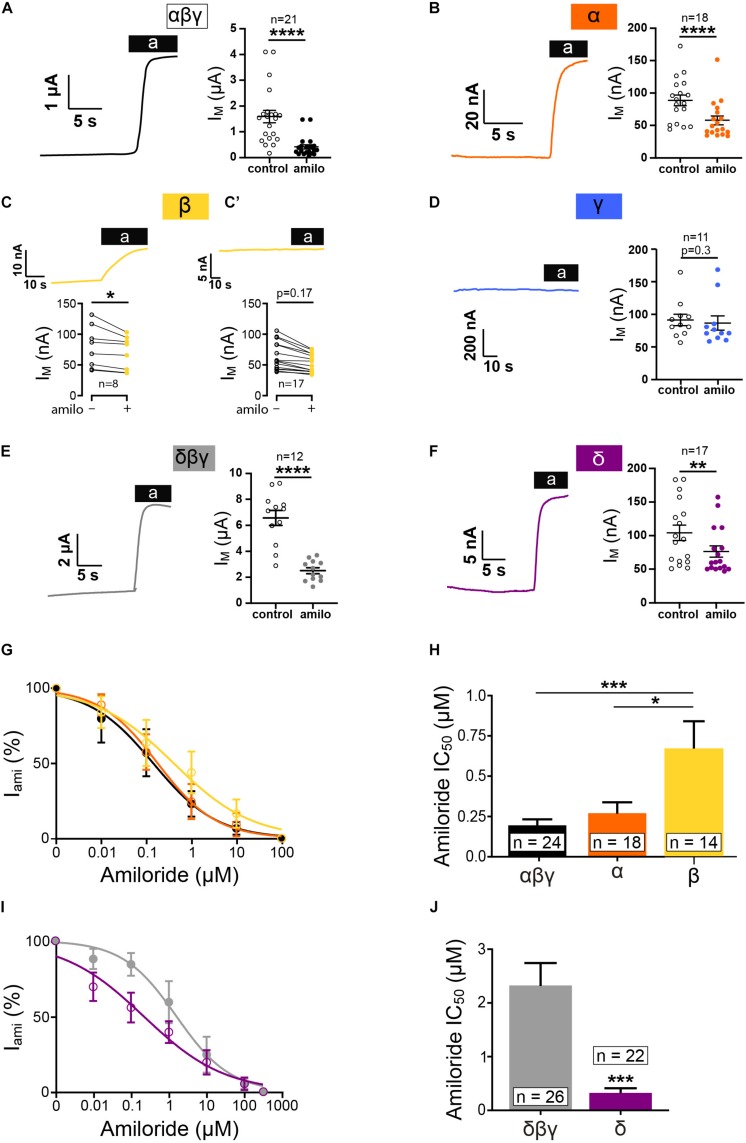
Homotrimeric ENaC forms functional channels except for γ. Electrophysiological characterization of homotrimeric ENaC (α, δ, β, and γ) vs. canonical ENaC (αβγ and δβγ) expressed in oocytes. Representative current traces of αβγ- **(A)**, α- **(B)**, β- **(C)**, γ- **(D)**, δβγ- **(E)**, and δ-injected oocytes **(F)** in response to the application of amiloride (10μM). **(C)** Oocytes injected with ENaC encoding RNA revealed variable responses. Only a proportion of oocytes responded to amiloride, whereas the majority of oocytes did not show an amiloride response **(C’)**. **(D)** Oocytes injected with γ ENaC did not show a reaction in response to amiloride. **(G)** Amiloride dose response curve of αβγ, α, and β ENaC and calculated IC_50_ values for amiloride **(H)** showing a decreased amiloride sensitivity for β when compared with αβγ, whereas α is unchanged. **(I)** Amiloride dose response curves of δβγ and δ. **(J)** δ ENaC displays a significantly reduced amiloride IC_50_ value compared with δβγ. Paired *t*-test **(A–F)**, one-way ANOVA with multiple comparison **(H)** and unpaired *t*-test **(J)**; *p < 0.05; **p < 0.01; ***p < 0.001; ****p < 0.0001.

To determine whether or not individual ENaC subunits can form functional homomeric ion channels, preliminary experiments were performed in *Xenopus* oocytes injected with mRNA encoding one subunit (either α, β, γ, or δ). Currents of heterotrimeric αβγ or δβγ ENaCs were measured in corresponding oocyte batches for comparison. ENaC-mediated currents were determined through the application of amiloride (10 μM). The expression of single subunits produced small but robust amiloride-sensitive currents for α and δ ENaC (Figure 1). In oocytes expressing β ENaC, only a proportion of oocytes showed an amiloride response (Figure 1C), and data were separated in two groups, responder and non-responder. Responders were identified by a deflection of current upon amiloride application as exemplified in the corresponding current trace in Figure 1C. Although in non-responder a decline in current was also observed in some cases (Figure 1C’). This decline was not caused by amiloride, and it represents a steady drift in baseline current and is likely caused by small leak currents and/or changing electrodes (offset potentials).

Similar to the β non-responder, no amiloride effects were detected following the injection of γ ENaC alone (Figure 1D) as well as in oocytes that were injected with water (Figure 3A). It may be noted that the amiloride-sensitive currents for α, β, and δ were only ∼2–10% in size compared with the corresponding heterotrimers (Figures 1A,E).

In addition, amiloride dose-response experiments were performed, and the changes in current to various amiloride concentrations were fitted with a Hill equation (Figure 1G). While the relative amiloride responses between αβγ and a were similar, α right shift was observed for β ENaC (here, only data from oocytes that were responsive to amiloride were used for analyses). Accordingly, the IC_50_ value for homomeric β ENaC was elevated (Figure 1H). Dose-response experiments with δβγ and δ ENaC revealed that the expression of δ ENaC alone does provide channels that have a higher affinity to amiloride in comparison to the heterotrimer (Figures 1I,J).

So far, these experiments indicate that, with the exception of γ ENaC, all tested subunits can form amiloride-sensitive homomeric channels in *Xenopus* oocytes.

**FIGURE 2:**
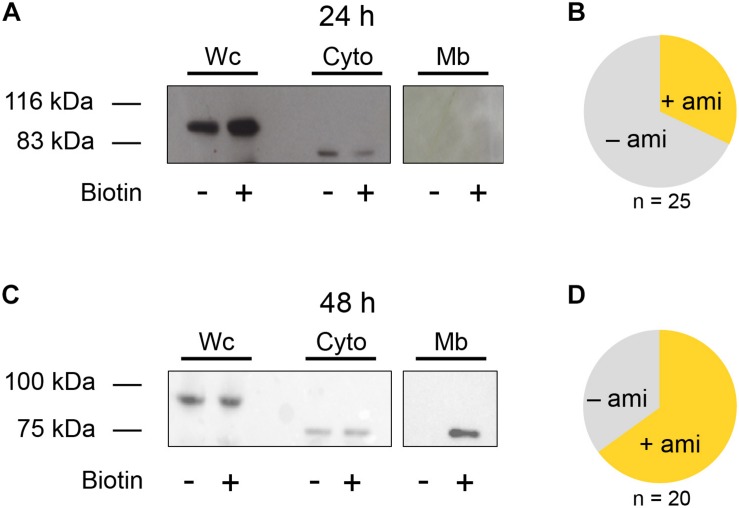
Biotinylation assay of β ENaC expressing oocytes. **(A)** Representative western blot showing β ENaC in presence (+) or absence (−) of biotin. Each group consists of the following three samples: whole cell (Wc), cytoplasmic (Cyto), and membrane bound (Mb). Exposure time for Wc and Cyto: 5 s and for Mb: 10 min *N* = 3. After an incubation of 24 h β ENaC was present in the Wc and Cyto fractions but absent in the Mb sample. It may also be noted that the Cyto and Mb fraction were treated with PNGase F, resulting in a lower molecular weight compared to the band detected in the Wc sample. **(B)** Fraction of β ENaC expressing oocytes exhibiting amiloride-sensitive currents (+ami) after 24 h incubation. **(C)** Increasing the incubation time of β ENaC to 48 h shows a clear band within the Mb fraction and increases the number of oocytes exhibiting amiloride-sensitive currents **(D)**.

Because the detection of amiloride-sensitive currents with β ENaC was inconsistent, the question arose if this is due to low protein abundance in the membrane or the inability to form functional channels in the membrane. To address this question, the β ENaC subunit was studied further. A biotinylation assay was performed to detect the membrane fraction (Mb) of β ENaC. A Flag-tagged β (β_Flag_) subunit was used for blotting to separate the membrane and cytosolic fraction (Cyto) at 24 and 48 h after injection of the mRNA. The Mb and Cyto fractions were also treated with PNGaseF, resulting in a band at a lower molecular weight representing a de-glycosylated β subunit. In accordance with a low number of oocytes showing an amiloride response (Figure 2B), only an insignificant amount of β_Flag_ ENaC could be detected 24 h after mRNA injection within the membrane fraction (Mb, Figure 2A). In contrast to this, the whole cell (Wc) and cytoplasmic (Cyto) fractions yielded a robust expression (Figure 2A, original blots included as Supplementary Material).

However, 48 h after injection, the proportion of amiloride-responsive cells was increased (Figure 2D), and this was accompanied by a clear β_Flag_ ENaC band detected in the Mb. This observation indicates a time-dependent increase of β ENaC in the membrane (Figure 2C, original blots included as Supplementary Material) and that the ability of β ENaC to form amiloride-sensitive channels is likely affected by its transport and insertion to the membrane, rather than to function.

### Homotrimeric α, β, and δ ENaC Can Be Activated by SF

To verify whether or not homotrimeric ENaC can respond to SF, oocytes expressing either α, β, γ, or δ ENaC were placed in a custom-made flow chamber and exposed to 0.2 dyn^∗^cm^–2^ SF via a pressurized perfusion system. The amiloride-sensitive current in absence of SF is indicated with I_0_, whereas the amiloride-sensitive current with 0.2 dyn^∗^cm^–2^ SF is labeled with I_0_._2_. For analyses, amiloride-sensitive currents before and after SF were compared.

**FIGURE 3:**
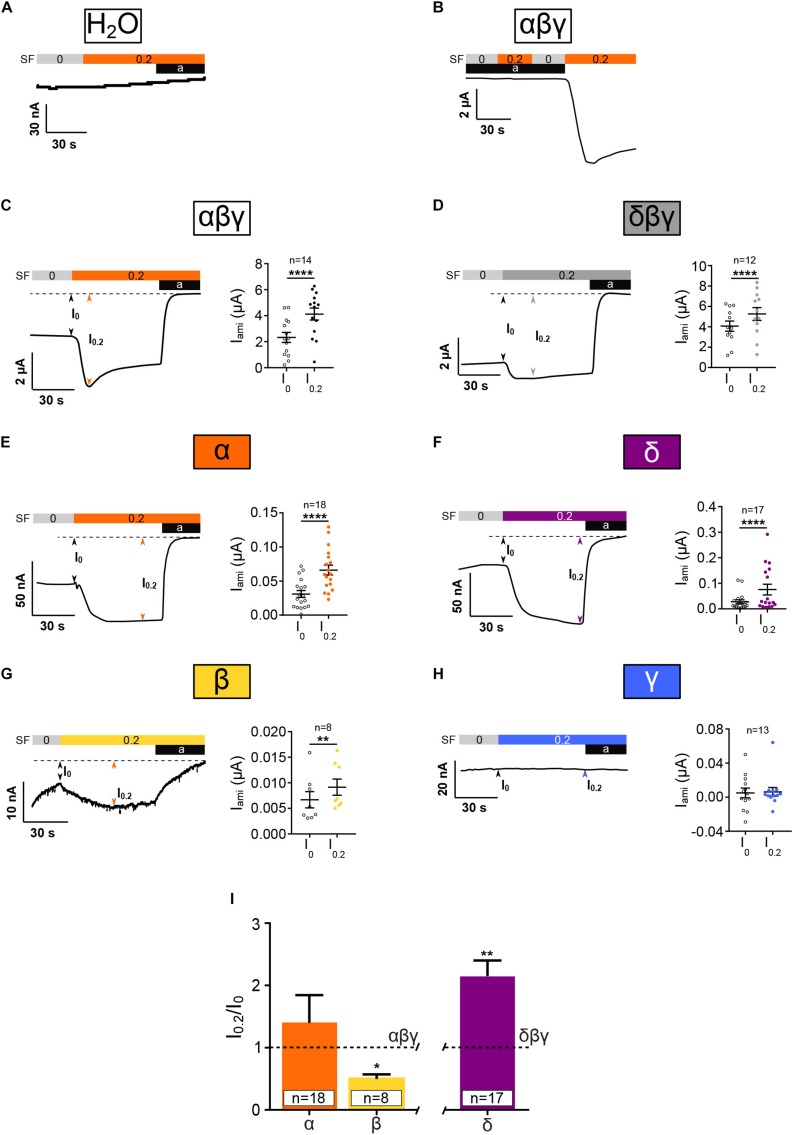
Homotrimeric ENaC except for γ is activated by SF. Electrophysiological characterization of homotrimeric ENaC in response to 0.2 dyn*cm^– 2^ SF (colored bar). **(A)** H_2_O-injected control oocytes neither respond to SF nor amiloride (10 μM). **(B)** SF response of αβγ ENaC is absent in presence of amiloride (10 μM). αβγ **(C)** and δβγ **(D)** ENaC are significantly activated by SF. Left panels show representative traces. The right panels show the averages ± SEM. Homotrimeric α, δ, and β (**E–G**, respectively) respond to SF, whereas γ **(H)** does not. **(I)** The SF response (I_0_._2_/I_0_) of homotrimeric ENaC subunits was normalized with respect to canonical αβγ and δβγ ENaC. The SF response of α ENaC was unchanged while β ENaC showed a reduced SF effect when compared with αβγ. δ ENaC displayed an increased SF effect compared with canonical δβγ ENaC. Paired *t*-test **(C–H)** and one-way ANOVA with multiple comparison **(I)**; *p < 0.05; **p < 0.01; ****p < 0.0001.

To verify whether changes in membrane current in response to SF are ENaC-mediated, water injected oocytes were exposed to amiloride and SF (Figure 3A). Also, αβγ ENaC expressing oocytes were exposed to SF in the presence of amiloride (Figure 3B). With both approaches, no SF effects were observed, indicating that active ENaC is needed to observe an SF response.

In accordance with previous studies, αβγ and δβγ ENaC were activated by the application of SF (Figures 3C,D). In oocytes that expressed homomeric α, β, or δ ENaC, a significant increased current was observed with 0.2 dyn^∗^cm^–2^ SF (Figures 3E–G). In contrast, the expression of γ ENaC alone did not yield an increased current in response to SF (Figure 3H). Thus, α, β, and δ ENaC subunits can form SF-sensitive homomeric ion channels, whereas γ ENaC alone does neither show amiloride-sensitive nor SF-activated currents.

In order to compare the relative responses of ENaCs formed by one subunit to SF a retrospective analysis was performed comparing the normalized SF effects (Figure 3F). SF currents (I_0_._2_) were normalized with respect to currents before SF (I_0_) was applied (I_0_._2_/I_0_). This is expected to adjust for the differences in amiloride-sensitive current amplitudes between canonical ENaC and homomeric ENaCs. I_0_._2_/I_0_ of canonical ENaCs (αβγ or δβγ) was defined as 1.0 ± SEM. The normalized SF effect of α ENaC was similar to αβγ ENaC, whereas the response of β was reduced and δ was elevated with respect to the corresponding heterotrimers (Figure 3I).

### Heterotrimeric ENaC Composed of Two Different Subunits Display Reduced Membrane Currents

**FIGURE 4:**
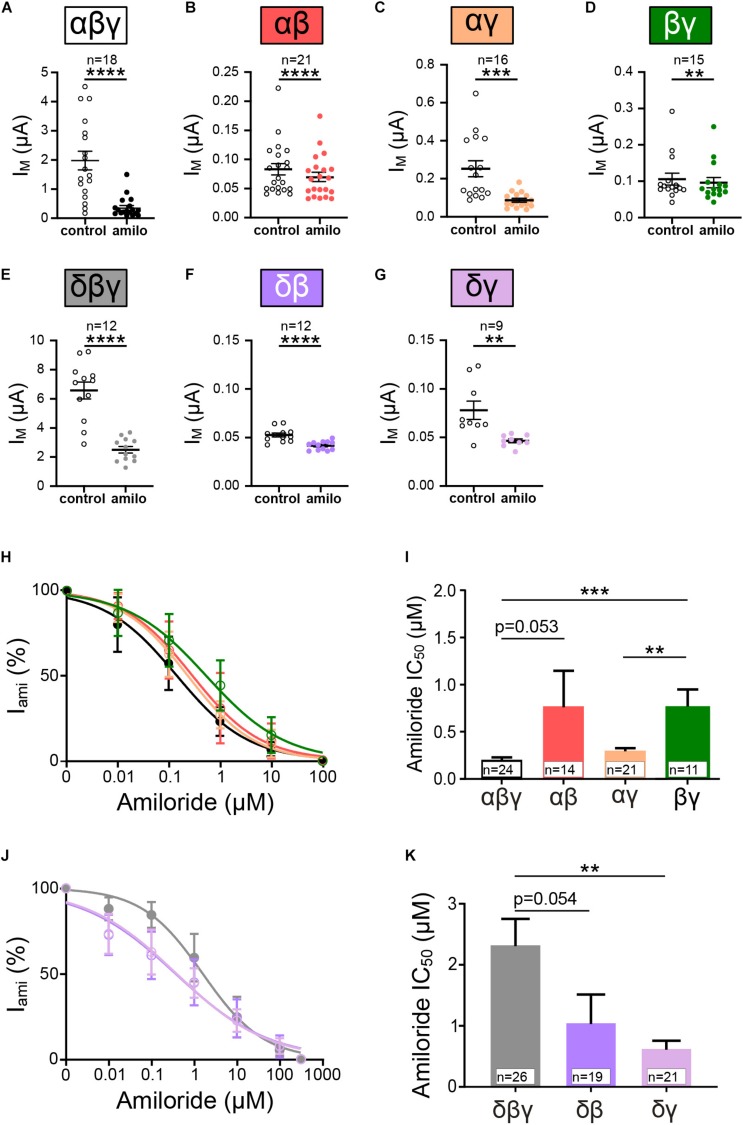
Heterotrimeric ENaC composed of two subunits form functional channels. **(A–G)** Effect of amiloride (10 μM) application on heterotrimeric ENaC currents of various subunit combinations (αβγ, αβ, αγ, βγ, δβγ, δβ, and δγ). **(H)** Dose response curves for amiloride were fitted and derived IC_50_ values were used for comparison **(I)**. Amiloride sensitivity of αβ and αγ ENaC was statistically not different when compared with αβγ, whereas βγ ENaC displays a significantly increased IC_50_ value with respect to αβγ and αγ ENaC. **(J)** Dose-response curves for channels containing δ ENaC. **(K)** δγ displayed a significantly reduced amiloride IC_50_ value compared with δβγ, whereas δβ remains unchanged. Paired *t*-test **(A–G)** and one-way ANOVA with multiple comparison **(I,K)**; **p < 0.01; ***p < 0.001; ****p < 0.0001.

In another approach channel combinations formed by two subunits (αβ, αγ, δβ, δγ, and βγ) were expressed and resulting currents measured. Similarly, as for the homomeric channels, amiloride-sensitive currents and SF responses were determined and compared with heterotrimeric αβγ and δβγ ENaC. All tested ENaC subunit compositions showed a significantly decreased current in response to amiloride application (Figures 4A–G). However, it may be noted that the presence of β ENaC in channels formed by two subunits seems to coincide with relatively low amiloride-sensitive currents in comparison with channels containing the γ subunit when co-expressed with α ENaC. The IC_50_ values for amiloride where unchanged for αβ and αγ when compared with canonical αβγ (Figures 4H,I). ENaC formed by αβ subunits displays a trend toward an increased IC_50_ value for amiloride, and the combination of βγ shows a significantly increased IC_50_ (Figures 4H,I).

Amiloride-sensitivity for channels including δ ENaC displayed contrary results. Here, the co-expression with γ ENaC resulted in a lower IC_50_ value (Figures 4J,K), indicating increased amiloride sensitivity, whereas IC_50_ values for δβ were unchanged (Figure 4K). Thus, ENaCs composed of variations of two different subunits display amiloride-sensitive currents, and the combination of subunits seem to modulate the affinity of amiloride to inhibit the channels.

### Heterotrimeric ENaCs Composed of Two Different Subunits Respond to SF

**FIGURE 5:**
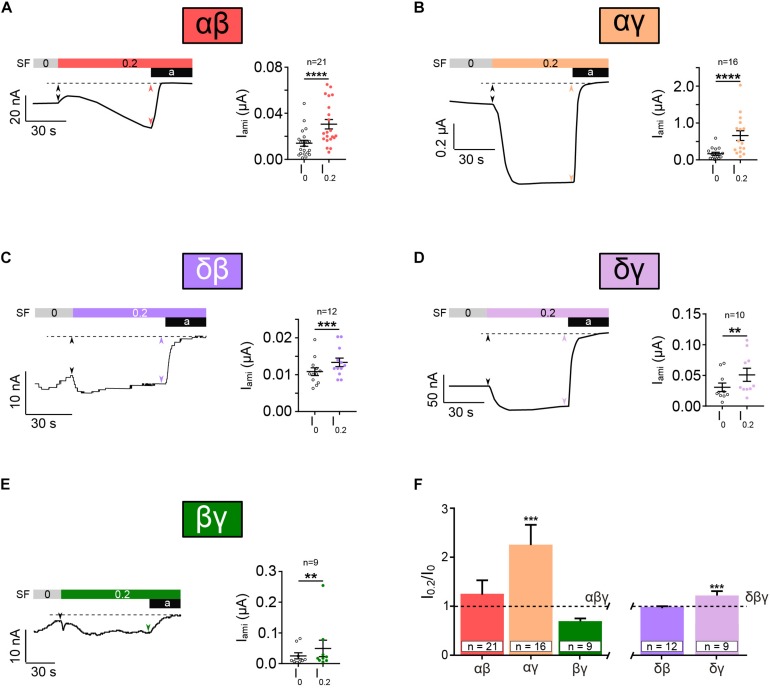
Heterotrimeric ENaC composed of two subunits responds to SF. SF response of heterotrimeric ENaC composed of two different subunits was assessed. Left panels show representative current traces whereas the right panels show the averages ± SEM. All combinations of two different ENaC subunits, αβ **(A)**, αγ **(B)**, δβ **(C)**, δγ **(D)**, and βγ **(E)**, were significantly activated by the application of SF (indicated as colored bar). **(F)** The SF response (I_0_._2_/I_0_) of those ENaC subunit compositions was normalized with respect to canonical αβγ and δβγ ENaC. The SF effect of αβ and βγ was unchanged when compared with αβγ. A significantly increased SF response was observed for αγ when compared with αβγ. δγ ENaC shows also a significantly increased SF effect when compared with δβγ, whereas δβ was unchanged. Paired *t*-test **(A–E)** and one-way ANOVA with multiple comparison **(F)**; **p < 0.01; ***p < 0.001; ****p < 0.0001.

Next, we examined whether or not ENaC formed by combinations of two subunits (αβ, αγ, δβ, δγ, and βγ) respond to the application of 0.2 dyn^∗^cm^–2^ SF. Surprisingly, all combination did show a significant increased current in responds to SF (Figures 5A–E). Here, as indicated by the amiloride-sensitive currents, the presence of β ENaC was associated with low SF responses (Figures 5A,C). This was in contrast to the observations when γ ENaC was present (Figures 5B,D), where SF responses were higher. Overall these experiments demonstrate that the principal ability of ENaC to respond to SF does not depend on the presence of three different subunits forming a trimer. It also indicates that the ability of ENaC to respond to SF is not a unique feature of canonical αβγ ENaC.

The comparison of the normalized SF responses reveals that αγ and δγ show a significantly increased SF response in comparison with αβγ and δβγ, respectively (Figure 5F). This indicates a potential role for γ ENaC as a “positive” modulator of SF responsiveness.

Taken together, this data shows that non-canonical ENaCs, except for γ, respond to SF. Additionally, the presence of γ ENaC seems to have a modulatory role in SF sensation, resulting in channels that are more responsive.

## Discussion

Heterotrimeric αβγ ENaC is a mechanosensitive ion-channel that responds to SF. This was shown in various expression systems (Carattino et al., 2004; Althaus et al., 2007; Karpushev et al., 2010), as well as in endothelial cells (Wang et al., 2009; Guo et al., 2016) and in native tissues such as the kidney epithelium (Satlin et al., 2001; Morimoto et al., 2006) and arteries (Ashley et al., 2018). Research on identifying how ENaC senses mechanical force usually focuses on canonical αβγ ENaC. However, the role of individual ENaC subunits for the ability of ENaC to respond to SF is unknown. This is of particular interest because previous studies show that in some tissues that are known to be involved in mechanosensation, either β and/or γ ENaC is expressed in absence of α ENaC (Drummond et al., 1998, 2000; Drummond, 2012). However, whether homotrimeric ENaCs or channels composed of only two different subunits form functional channels that respond to SF remains unknown and was a key aim addressed in this study.

### Homotrimeric α, δ, or β ENaCs Are Activated by Shear Force

Evidence that homomeric ENaCs are functional was provided by a study of Canessa and colleagues by detecting amiloride-sensitive currents following the expression of individual subunits in *Xenopus* oocytes (including β and γ; Canessa et al., 1994b). Our results are in agreement with this early observation. Furthermore, Canessa et al. (1994b) showed the amiloride-sensitive currents obtained with single subunits are small in comparison with the corresponding heterotrimeric channels formed by expression of either αβγ or δβγ-ENaC. The reason for the small currents was previously identified to be due to a reduction in membrane expression of homotrimeric ENaCs (Firsov et al., 1996). This is also in agreement with our observation with regard to the surface expression of β ENaC. Here, the number of oocytes showing an amiloride response as a measure of ENaC function was increased after 48 h of incubation. This observation was also underpinned by a time-dependent increased amount of β ENaC protein at the surface of the oocytes (Figure 2). Similarly, time-dependent expression of βγ ENaC was reported in *Xenopus* oocytes (Bonny et al., 1999). Interestingly, we were not able to detect amiloride-sensitive currents in oocytes expressing γ ENaC alone. This is also supported by previous observation that γ ENaC was undetectable in the membrane of oocytes when expressed alone (Firsov et al., 1996).

Amiloride IC_50_ values of homotrimeric β ENaC were elevated. It may be hypothesized that this reduced amiloride affinity accounts for the variability of results reported for amiloride effects in arteries involving “non-classical ENaC” channels (Perez et al., 2009). The study of Perez and colleagues reported that the effects they observed were more sensitive to amiloride in comparison to benzamil. Commonly, benzamil is known to have a higher affinity for canonical ENaC and thus indicates the existence of non-canonical ENaCs in arteries. To reveal if these non-canonical ENaCs could be formed by β ENaC subunits, it may be considered to perform benzamil dose-response experiments and compare IC_50_ values with those of amiloride. Expression of homotrimeric δ ENaC revealed an increased affinity to amiloride in comparison with δβγ ENaC, indicating that the presence of other subunits may cause the decreased amiloride affinity in comparison with αβγ ENaC. Whether or not homotrimeric ENaC channels are relevant in native cells remains unknown. Evidence suggests that in *Xenopus* oocytes the membrane abundance of single subunits is impaired. It may be hypothesized that the efficiency of ENaC subunits being transported and inserted into the membrane depends on suitable machinery. It may be speculated that other cell types may be more suitable for delivering homotrimeric ENaCs to the membrane.

As far as we are aware, this is the first study investigating how homotrimeric channels respond to SF. With the exception of γ ENaC, all homomers were activated by SF. In accordance with the low amiloride-sensitive currents, the SF responses were small. However, the normalized SF responses (Figure 3I) indicate that β ENaC is less responsive to SF when compared with αβγ ENaC, and δ ENaC is more strongly activated by SF when compared with δβγ ENaC. This indicates that the subunits and their interaction could influence the ability of the channel to respond to SF. Inter-subunit cytoplasmic interactions are important in modulating gating of ENaC (Berdiev et al., 2000), and this may also contribute to SF activation. A limitation of this comparison is represented by the use of one dose of amiloride only. Channels with an elevated IC_50_ value (as determined in the dose-response curves) may not be fully inhibited by the use of 10 μM amiloride, and this may affect the normalized currents used for this comparison. It may also be noted that to reveal whether or not single subunits are more (or less) sensitive to SF was not a major focus if our present study and requires future experiments (SF dose-responses) for conclusive results. Nevertheless, a major observation of the present experiments is that, with the exception of γ ENaC, homomeric α, β, and δ ENaC channels can respond to SF.

### ENaCs Formed by Two Subunits Are Activated by Shear Force

The responses to amiloride that we have observed were less variable in different oocytes compared with homotrimeric channels (e.g., β ENaC). For all tested subunit combinations, significant effects with amiloride were observed, which is in agreement with previous reports (Canessa et al., 1994a; Bonny et al., 1999). In addition, all subunit combinations were able to show increased currents in response to SF.

Comparing the normalized SF responses revealed that the combination of αβ ENaC yielded responses that were similar in comparison to αβγ. In contrast, αγ ENaC yielded a proportionally elevated SF response, and this also coincided with an increased amiloride-sensitive current in comparison to αβ ENaC. This observation may be supported by previous studies showing that the γ subunit is important for channel trafficking (Konstas and Korbmacher, 2003). Taking into account that the γ subunit was not able to form channels by itself, it seems that the γ ENaC plays an important role for promoting ENaC function by facilitating membrane expression and SF activation.

### Physiological Relevance of Modulatory Role of the β or γ ENaC Subunit in Humans

The different combinations of ENaC subunits influencing the channels ability to sense SF may provide new insights into our understanding of mechanosensory processes in the kidney and the vasculature. Here, ENaC is exposed to SF rates from approximately 0.2–20 dyn^∗^cm^–2^ in the kidney (Cai et al., 2000) and 20–40 dyn dyn^∗^cm^–2^ in the vasculature (Davies, 1995). Potential changes of subunit expression in the membrane to adapt to changes in the mechanical environment might provide new perspectives for the understanding of normal physiological and pathophysiological processes contributing to blood pressure regulation in the kidney and the vasculature.

Such a potential change of subunit composition might be a common feature of cells to form ion channels with distinct functional characteristics. This is in agreement with previous studies providing evidence for organ/tissue-specific expression patterns of ENaC subunits. For example, in rodent lungs, high α and γ ENaC mRNA expression was detected with little or no β ENaC (Farman et al., 1997), as well as increased expression of α and γ to compensate for the loss of β ENaC (Randrianarison et al., 2008). Accordingly, it may be suggested that the majority of channels in such situations are presumably ααγ or αγγ ENaCs rather than canonical αβγ ENaC. Channels formed by ag subunits seem to protect mice lacking β ENaC from respiratory failure (McDonald et al., 1999), indicating that these two subunits provide sufficient function at the organ level. Based on our finding, one might further speculate that the main function of such channels in lungs is related to the ability to effectively sense and respond to mechanical forces. Considerable support for the role of non-canonical ENaC as mechanosensors derives from observations that β and γ ENaC are expressed in the baroreceptors and nerve endings of rat foot pad and vascular smooth muscle cells in the absence of α ENaC (Drummond et al., 1998, 2000, 2001, 2004; Grifoni et al., 2006). Furthermore, there is evidence for reduced expression of β and γ ENaC in aortic baroreceptors that coincided with baroreceptor dysfunction in a rat model of chronic heart failure (Li et al., 2016). Interestingly, non-canonical ENaC subunit combination was also reported in dentritic cells to mediate inflammatory responses (Barbaro et al., 2017). These reports support the idea that in certain tissues and cells only one or two types of ENaC subunits are expressed that can form a functional channel. Further, a recent study indicates the role for ENaC as flow sensor being important for the proliferation of neuronal stem cells (Petrik et al., 2018). This study only detected α ENaC in these cells supporting a potential role of single ENaC subunits as local mechanosensors. In the kidney and vasculature, such non-canonical ENaCs can contribute to blood pressure regulation that is dependent on the ability to sense mechanical force and SF in particular. Last but not least, it also has to be taken into account that there is evidence for the presence of ENaC subunits and acid sensing ion channels (ASICs) subunits in baroreceptors (Drummond et al., 2001; Lu et al., 2009) and vascular smooth muscle cells (Grifoni et al., 2006, 2008). This raises the possibility that ENaC and their related ASIC proteins (Mano and Driscoll, 1999) may complex to from non-canonical cation channels. This possibility is strongly supported by a study providing evidence for the function of a non-selective cation channels formed by ENaC and ASIC subunits (Trac et al., 2017). These observations indicate that in tissues where only one or two ENaC subunits were identified, these may not only be able for form functional non-canonical ENaC channels but also associate with ASIC subunits and form ENaC/ASIC complexes. Such ENaC/ASIC complexes may be characterized by altered inhibitory profile (e.g., low benzamil affinity) and lower amiloride affinity in comparison with canonical ENaC. This will further increase possibilities for channel compositions and stoichiometry.

## Conclusion

In conclusion, we found that homotrimeric ENaC, except for γ, as well as heterotrimeric ENaC composed of only two different subunits form functional channels with low amiloride-sensitive currents that respond to SF. In combination with other subunits, the γ ENaC subunit seems to facilitate robust SF responses. Hence, this modulatory role of subunit composition and the resulting SF sensitivity can have new, yet unidentified physiological roles in mechanosensation processes.

## Data Availability Statement

The datasets generated for this study are available on request to the corresponding author.

## Ethics Statement

The animal study was reviewed and approved by theAnimal Ethics Committee of the University of Otago.

## Author Contributions

J-PB and DB performed the experiments, analyzed data, and drafted the manuscript. MF led the project and finalized the manuscript. All authors contributed to the design of the work, the analysis, and interpretation of the data. All authors agreed to be accountable for the content of the work.

## Conflict of Interest

The authors declare that the research was conducted in the absence of any commercial or financial relationships that could be construed as a potential conflict of interest.
