# Supplementary figures and images for: Epithelial Na+ Channel (ENaC) Formed by One or Two Subunits Forms Functional Channels That Respond to Shear Force
Source: Front Physiol. 2020 Mar 17;11:141. doi: 10.3389/fphys.2020.00141 (PMC7090232; doi:10.3389/fphys.2020.00141)

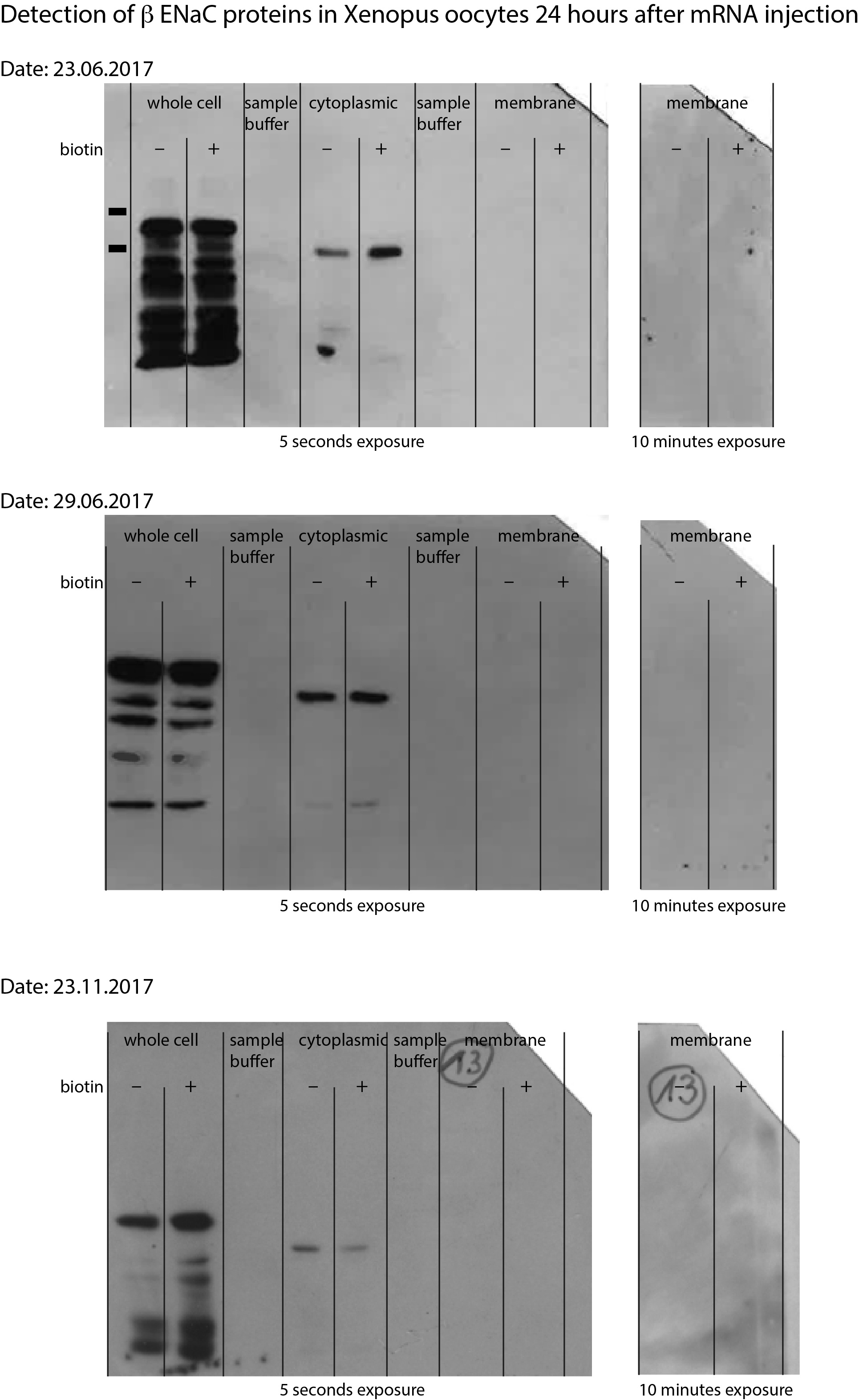

Supplement: Supplementary file 1 [file Image_1.JPEG]

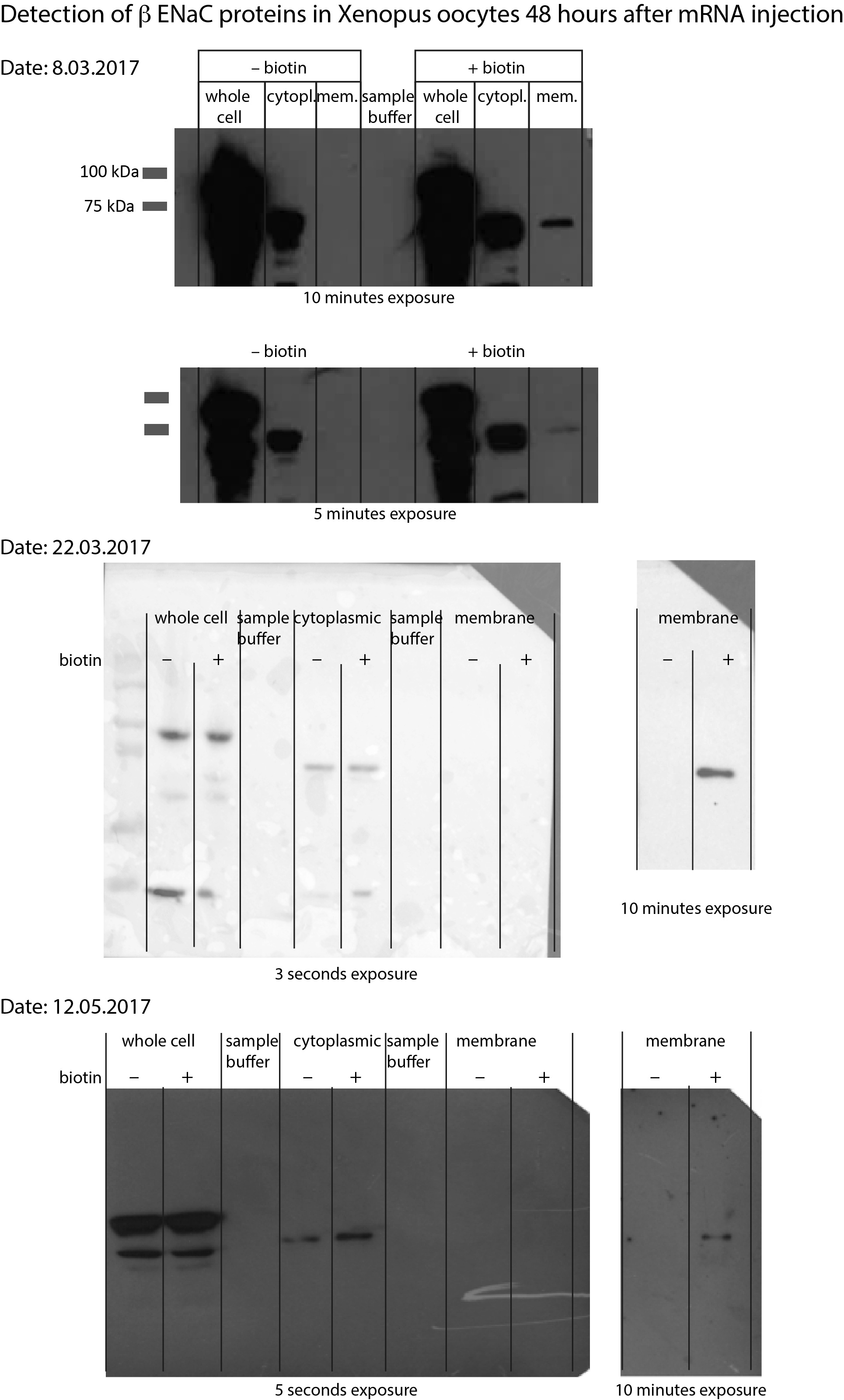

Supplement: Supplementary file 2 [file Image_2.JPEG]
